# Supplementary material for: Kif11-haploinsufficient oocytes reveal spatially differential requirements for chromosome biorientation
Source: EMBO Rep. 2025 Aug 20;26(18):4419–35. doi: 10.1038/s44319-025-00539-w (PMC12457643; doi:10.1038/s44319-025-00539-w)
Supplement: Supplementary file 2 — Movie EV1 [file 44319_2025_539_MOESM2_ESM.zip › Movie_EV1_README.docx]

**Movie EV1: KIF11 dose-dependent differences in spindle elongation.**

Live imaging of spindle shape dynamics during meiosis I. Z-projection images of microtubules (EGFP-MAP4, green) and chromosomes (H2B-mCherry, magenta) are shown. Time in hh:mm. Scale bar, 5 μm. See also Fig. 2.
