# Supplementary material for: Kif11-haploinsufficient oocytes reveal spatially differential requirements for chromosome biorientation
Source: EMBO Rep. 2025 Aug 20;26(18):4419–35. doi: 10.1038/s44319-025-00539-w (PMC12457643; doi:10.1038/s44319-025-00539-w)
Supplement: Supplementary file 3 — Movie EV2 [file 44319_2025_539_MOESM3_ESM.zip › Movie_EV2_README.docx]

**Movie EV2: Difference in the spindle bipolarization in response to the dose of KIF11.**

Live imaging of spindle bipolarity dynamics during meiosis I. Z-projection images of MTOCs (mNG-CEP192, green) and chromosomes (H2B-mCherry, magenta) are shown. Time in hh:mm. Scale bar, 5 μm. See also Fig. 3.
