# Supplementary material for: Kif11-haploinsufficient oocytes reveal spatially differential requirements for chromosome biorientation
Source: EMBO Rep. 2025 Aug 20;26(18):4419–35. doi: 10.1038/s44319-025-00539-w (PMC12457643; doi:10.1038/s44319-025-00539-w)
Supplement: Supplementary file 12 — Expanded View Figures [file 44319_2025_539_MOESM12_ESM.pdf]

Expanded View Figures

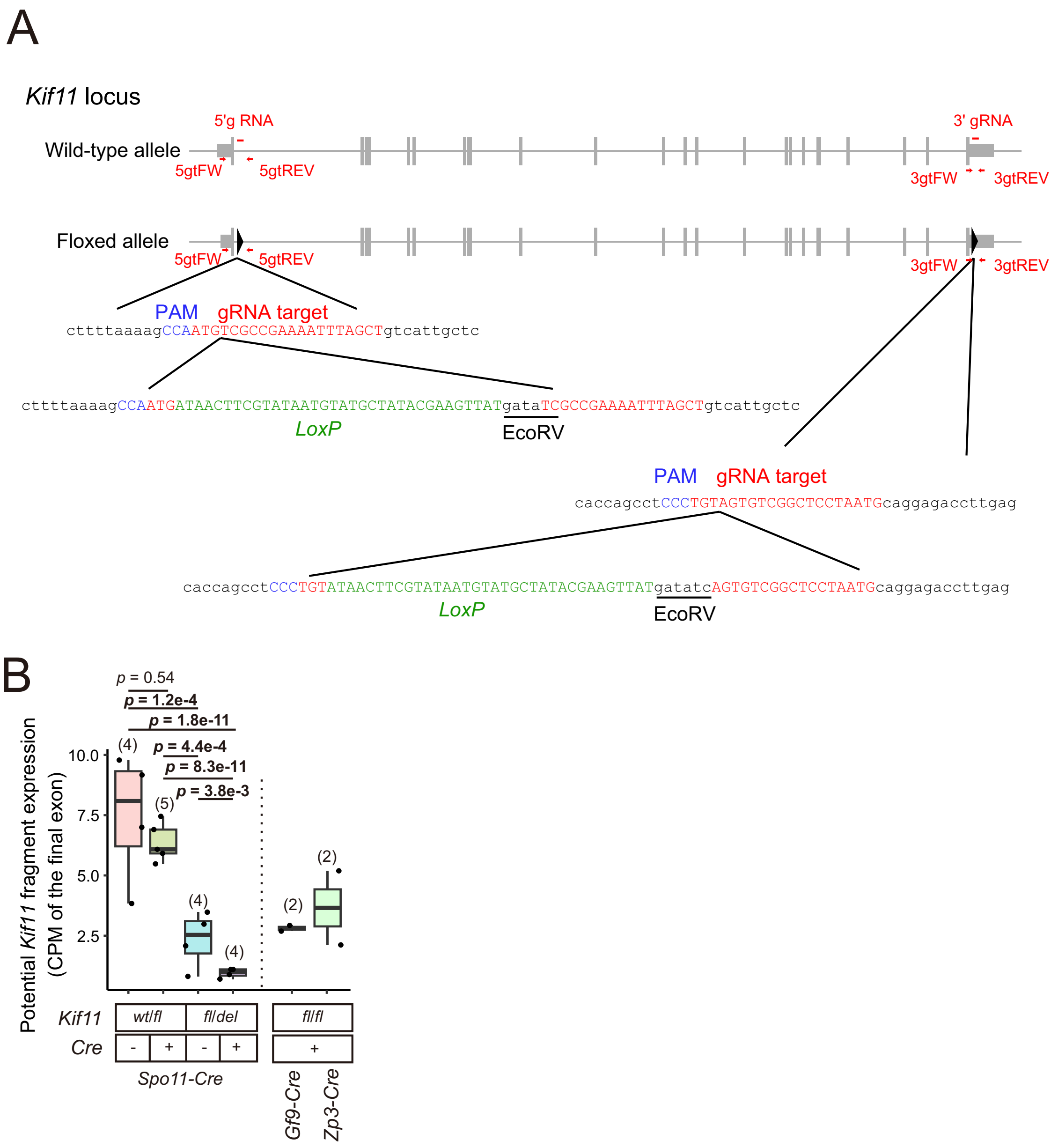

Figure EV1. Generation of *Kif11* floxed mice.

(**A**) Diagram of the *Kif11* gene locus. Thin exon segments denote untranslated regions (UTR), and the thick exon segments denote coding regions. Black triangles indicate target sites for CRISPR-Cas9-mediated *Loxp* (green) insertion. Red arrows indicate primer positions used for genotyping. The protospacer adjacent motif (PAM, blue) and the guide RNA (gRNA) target sequences (red) are marked. (**B**) Box plot comparing the relative expression levels of residual *Kif11* transcripts as measured by the coverage of the final exon from mice oocytes with respect to their genotype (Welch's *t*-test with Holm's correction for multiple comparisons; The numbers in parentheses above the box plot indicate the number of biologically independent experiments). Boxplots: Centerlines indicate the median; box limits represent the 25th and 75th percentiles; whiskers extend to the minimum and maximum values no further than 1.5 * IQR from the hinge (where IQR is the inter-quartile range, or distance between the first and third quartiles). Source data are available online for this figure.

© The Author(s)

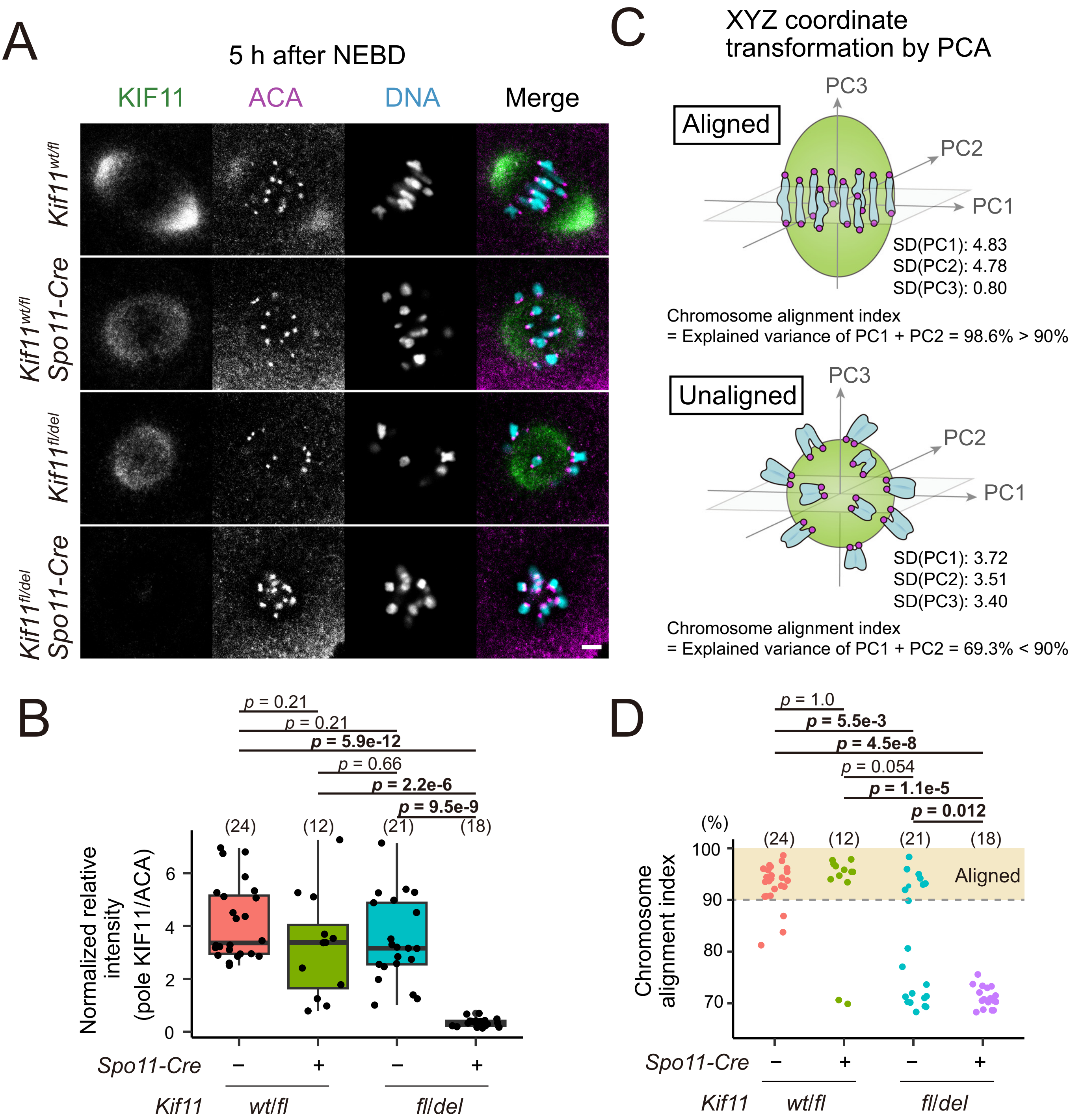

Figure EV2. Validation of KIF11 depletion.

(**A**) Oocytes were stained for KIF11 (green), ACA (kinetochores, magenta), and Hoechst 33342 (DNA, cyan) fixed at 5 h after NEBD (metaphase). Single z-section images are shown. Scale bar, 5 µm. (**B**) Quantification of KIF11 accumulated at the spindle pole. KIF11 signals in *Kif11$^{fl/del}$ Spo11-Cre* oocytes were quantified at the chromosome centers. Welch's *t*-test comparing all possible pairs with Holm's correction for multiple comparisons. (**C**, **D**) Chromosome alignment defects in *Kif11*-deleted oocytes. Chromosome alignment index, defined as the sum of explained variance of PC1 and PC2 from PCA-transformed coordinates of chromosome position in 3D images, greater than 90% were considered as "chromosome-aligned" oocytes. The statistically significant differences ($p < 0.05$) in the frequency of chromosome-aligned oocytes between genotypes were compared by Fisher's exact test comparing all possible pairs with Holm's correction for multiple comparisons. Two biologically independent experiments were performed for panels (**A**, **B**, **D**). Boxplots: Centerlines indicate the median; box limits represent the 25th and 75th percentiles; whiskers extend to the minimum and maximum values no further than 1.5 * IQR from the hinge (where IQR is the inter-quartile range, or distance between the first and third quartiles). Source data are available online for this figure.

 © The Author(s)

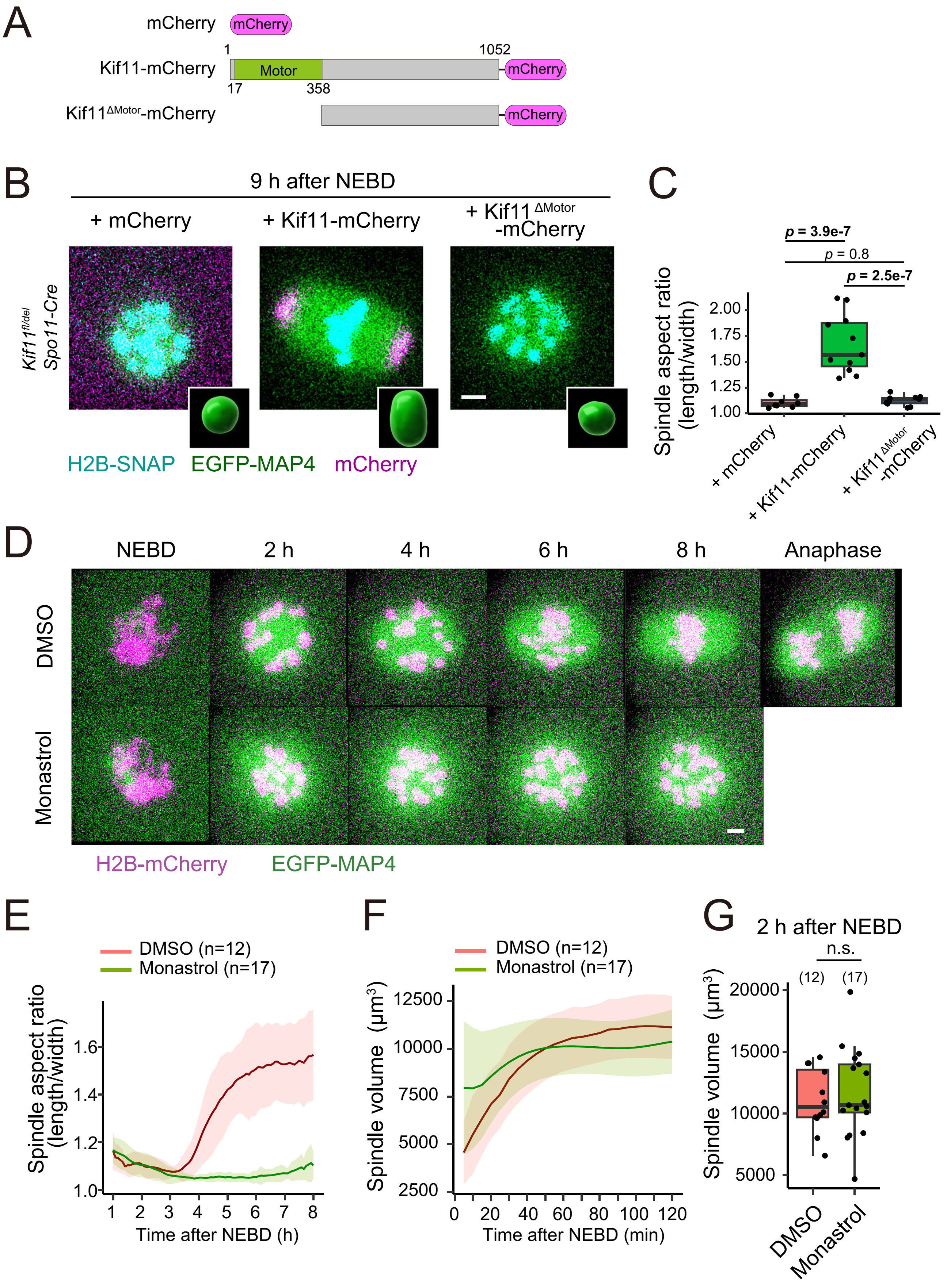
A
mCherry
mCherry
Kif11-mCherry
1
1052
Motor
17
358
mCherry
Kif11ΔMotor-mCherry
mCherry
B
9 h after NEBD
+ mCherry
+ Kif11-mCherry
+ Kif11 ΔMotor -mCherry
Kif11fl/del Spo11-Cre
H2B-SNAP
EGFP-MAP4
mCherry
C
p = 3.9e-7
p = 0.8
p = 2.5e-7
Spindle aspect ratio (length/width)
2.00
1.75
1.50
1.25
1.00
+ mCherry
+ Kif11-mCherry
+ Kif11 ΔMotor -mCherry
D
NEBD
2 h
4 h
6 h
8 h
Anaphase
DMSO
Monastrol
H2B-mCherry
EGFP-MAP4
E
DMSO (n=12)
Monastrol (n=17)
Spindle aspect ratio (length/width)
1.6
1.4
1.2
1.0
1 2 3 4 5 6 7 8
Time after NEBD (h)
F
DMSO (n=12)
Monastrol (n=17)
Spindle volume (µm3)
12500
10000
7500
5000
2500
0 20 40 60 80 100 120
Time after NEBD (min)
G
2 h after NEBD
n.s.
(12)
(17)
Spindle volume (µm3)
20000
15000
10000
5000
DMSO
Monastrol

Figure EV3. Rescue experiments and phenotypes caused by KIF11 inhibition with monastrol.

(**A**) Kif11 constructs used for rescue experiments. *Kif11*$^{\Delta Motor}$ lacks the motor domain. (**B**) Live imaging of *Kif11*$^{fl/del}$ *Spo11-Cre* (conditional homozygous deletion) oocytes expressing EGFP-MAP4 (microtubules, green), H2B-SNAP (chromosomes, cyan), and one of the Kif11-mCherry, Kif11$^{\Delta Motor}$-mCherry or mCherry (magenta). Z-projection images and 3D-reconstructed spindle images are shown. Scale bars, 5 µm. (**C**) Box plot comparing spindle elongation at metaphase (9 h after NEBD) (Welch's *t*-test comparing all possible pairs with Holm's correction for multiple comparisons). (**D**) Live imaging of BDF1 mouse oocytes expressing EGFP-MAP4 (microtubules, green) and H2B-mCherry (chromosomes, magenta) in the presence of DMSO (control) or monastrol. Z-projection images are shown. Scale bars, 5 µm. (**E**) Spindle elongation over time is shown as mean ± SD. The aspect ratio (length/width) of 3D-reconstructed spindles was measured. (**F**) Spindle volume with mean ± SD determined from 3D-reconstructed images. (**G**) Box plot comparing spindle volume at prometaphase (2 h after NEBD) between control (DMSO) and monastrol ($p = 0.63$, Welch's *t*-test). The numbers in parentheses above the box plot indicate the number of oocytes used for experiments. Three or two biologically independent experiments were performed for panels (**A**–**G**), respectively. Boxplots: Centerlines indicate the median; box limits represent the 25th and 75th percentiles; whiskers extend to the minimum and maximum values no further than 1.5 * IQR from the hinge (where IQR is the inter-quartile range, or distance between the first and third quartiles). Source data are available online for this figure.

 © The Author(s)

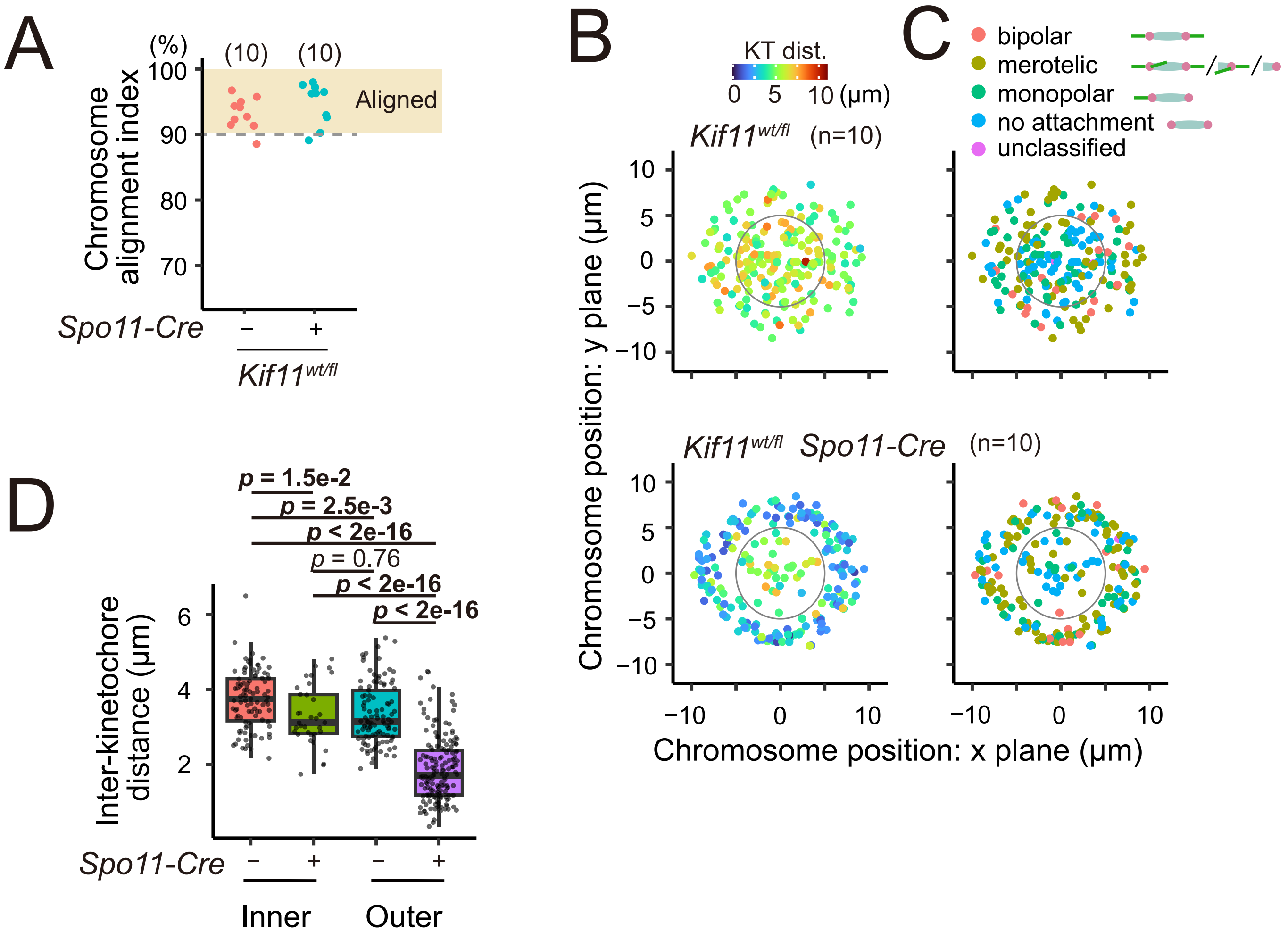

Figure EV4. Spatial difference of KT-MT attachment status with respect to chromosome stretching.

(**A**) Chromosome alignment index (see Fig. EV2) of oocytes analyzed for KT-MT attachments 5 h after NEBD. Each point represents one oocyte. The numbers in parentheses above the plots indicate the number of oocytes used for experiments. (**B**) Distribution of chromosome positions on the spindle equator with color codes indicating inter-kinetochore distance. (**C**) Chromosome positions are shown as in B, with color indicating KT-MT attachment status. (**D**) Box plot comparing inter-kinetochore distance with respect to chromosome position (inner or outer; see Fig. 4F) between genotypes. The statistically significant differences in inter-kinetochore distance between the chromosome positions were compared for each genotype by Welch's *t*-test for all possible pairs with Holm's correction for multiple comparisons. Three biologically independent experiments examining a total of ten oocytes for each genotype were performed. Boxplots: Centerlines indicate the median; box limits represent the 25th and 75th percentiles; whiskers extend to the minimum and maximum values no further than 1.5 * IQR from the hinge (where IQR is the inter-quartile range, or distance between the first and third quartiles). Source data are available online for this figure.

© The Author(s)
